# Surveillance of Norovirus in Nationwide Groundwater Sources in South Korea: A Comprehensive Five-Year Study

**DOI:** 10.3390/v16121814

**Published:** 2024-11-21

**Authors:** Jung Eun Lee, Jihye Kim, Jihyun Kang, Kyung Seon Bae, Eung-Roh Park, Jeong-Ki Yoon

**Affiliations:** Division of Water Supply and Sewerage Research, National Institute of Environmental Research, Incheon 22689, Republic of Korea; jihye4478@korea.kr (J.K.); jhkang2@korea.kr (J.K.); baeks1207@korea.kr (K.S.B.); erpark@korea.kr (E.-R.P.); jkyun@korea.kr (J.-K.Y.)

**Keywords:** norovirus, groundwater, norovirus genotype, water quality

## Abstract

Groundwater is an essential drinking water source for humans. However, improper groundwater management leads to fecal contamination and waterborne diseases caused by viral pathogens. Therefore, this study aimed to investigate norovirus (NoV) contamination by conducting nationwide monitoring over five years (2019–2023). Groundwater samples were analyzed for water quality parameters, indicator microorganisms, NoV prevalence, and viral genotypes. Water quality was assessed for temperature, turbidity, and residual chlorine, whereas microorganisms were analyzed for total coliforms, *Escherichia coli*, and NoV genotypes. Of the 600 sites, 11 (1.8%) were NoV-positive, irrespective of season or location. Low residual chlorine levels (0.02–0.75 mg/L) were observed, possibly limiting viral inactivation. Total coliforms were detected in only three NoV-positive samples, and *E. coli* was absent. NoV genotypes were identified as GI.1 and GII.4, with GII.4 being the most frequently detected genotype. The present study demonstrated that periodic monitoring and expanded nationwide efforts are required for effective groundwater management and public health protection.

## 1. Introduction

Gastroenteritis is a major public health concern worldwide and is associated with high morbidity and mortality rates. Noroviruses (NoVs) are the leading cause of acute non-bacterial gastroenteritis across all age groups, accounting for approximately 90% of viral gastroenteritis outbreaks worldwide [1,2]. In addition, the ability of NoVs to cause disease at extremely low infectious doses and its prolonged shedding period further complicate control efforts, making NoVs a formidable public health threat [3].

NoVs possess a single-stranded RNA genome and are classified into five genogroups (GI–GV), three of which infect humans (GI, GII, and GIV). NoVs are widely distributed in various environments. Numerous NoV outbreaks have been reported worldwide, with waterborne pandemics being particularly significant. Notably, a large proportion of these outbreaks has been attributed to NoV GI and GII [2]. NoVs are primarily transmitted through several routes, including contact with contaminated gastrointestinal secretions from infected individuals, ingesting contaminated water or food, person-to-person contact, and direct contact with contaminated surfaces [4]. NoVs transmitted through these routes can be detected in raw water, sewage, treated water, and drinking water and can potentially reach groundwater resources [5,6].

Groundwater is a crucial drinking, industrial, and domestic water source in developing and developed countries and is safer than surface water because of natural soil filtration, which reduces pathogen loads [7]. Groundwater is also widely available in many regions, is relatively easy and inexpensive to develop, and often requires minimal treatment before use [8,9]. In 2005, approximately 20% of all water withdrawals in the United States and 11% of all water supplies in South Korea were derived from groundwater [9]. However, improper groundwater management and exploitation have led to fecal contamination and waterborne diseases associated with viral pathogens, which have been reported worldwide. These outbreaks can result from direct consumption of contaminated water or food prepared using such water [5,10,11,12].

In South Korea, the largest foodborne disease outbreak associated with NoVs occurred in 2006, affecting approximately 2000 schoolchildren [13]. The outbreak was caused by food contaminated with NoV-infected groundwater [14]. Groundwater was analyzed for NoVs after the outbreak; however, these analyses have primarily been conducted sporadically and in a limited number of locations [9,15]. Therefore, the aim of this study was to investigate the safety of groundwater used as a drinking water source by conducting a five-year nationwide monitoring of 600 sites, assessing NoVs, microbial contamination levels, and overall water quality.

## 2. Materials and Methods

### 2.1. Sampling Sites

**Figure 1:**
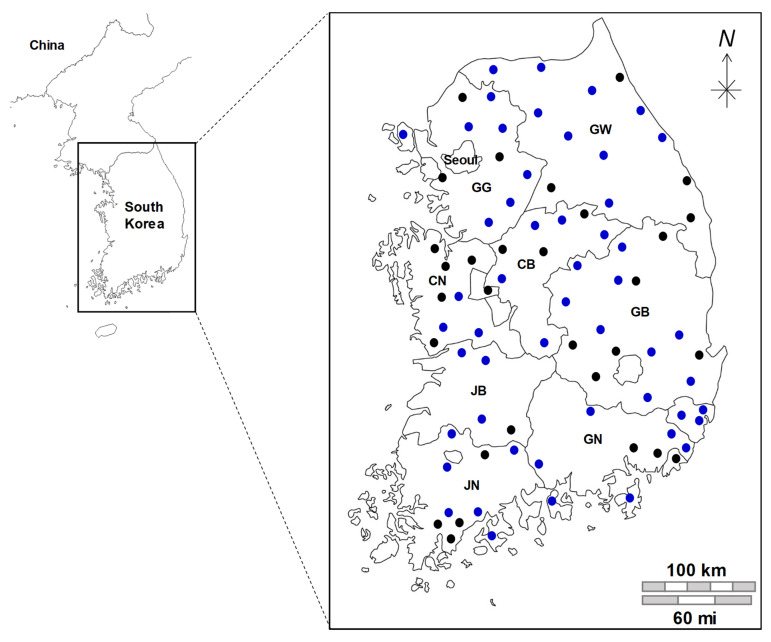
Sampling area for norovirus surveillance in South Korea. Black dots (●) represent areas sampled once, whereas blue dots (●) indicate areas that were sampled multiple times over five years. GG, Gyeonggi-do; GW, Gangwon-do; CB, Chungcheongbuk-do; CN, Chungcheongnam-do; GB, Gyeongsangbuk-do; GN, Gyeongsangnam-do; JB, Jeollabuk-do; JN, Jeollanam-do.

Samples from groundwater used as drinking water were collected from 600 distinct locations throughout South Korea. These locations are distributed across 83 counties and cities, representing the smallest administrative divisions for this analysis. This comprehensive sampling effort spanned five years, from February 2019 to November 2023. Notably, the same 83 cities or counties overlapped throughout the study period (Figure 1), and sampling was primarily performed during the spring, fall, and winter seasons.

### 2.2. Water Sampling and Processing

The sampling apparatus consisted of a flow meter, pressure gauge, Liquid Filter Cartridge (cat. JN2822006, ENVIONEER, Jecheon, Republic of Korea), cartridge filter housing, and tubing. The assembly was manufactured according to the U.S. Environmental Protection Agency (EPA) manual (method 1615). Samples were collected from tap water using groundwater at most sites. A sample was collected to measure water temperature, turbidity, and residual chlorine after flushing some of the water. Subsequently, over 500 L were collected from most of the sites. After sampling, the cartridge filter was removed from the housing, immediately stored at 4 °C, and concentrated according to U.S. EPA method 1615 within 72 h of sample collection [16]. Final concentrates were stored at −70 °C until analysis. Additional water samples were collected using sterile polypropylene bottles to analyze fecal indicator bacteria.

### 2.3. RNA Extraction and NoV Analysis

Viral RNA was extracted using the QIAamp Viral RNA Mini Kit (Qiagen, Hilden, Germany), according to the manufacturer’s instructions. All reverse transcription polymerase chain reaction (RT-PCR) amplification steps were conducted using a Qiagen OneStep RT-PCR Kit (Qiagen). Two previously reported RT-PCR and nested PCR assays were used to detect each NoV genogroup (GI and GII) [14,17]. Consequently, two RT-PCR and two nested PCR assays were executed for each sample. Primer sets for RT-PCR (GI-F1M/GI-R1M) and for nested PCR (GI-F2/GI-R1M) were used to analyze GI NoVs. Similarly, primer sets for RT-PCR (GII-F1M/GII-R1M) and nested PCR (GII-F3/GII-R1M) were used for GII NoV analysis. The RT-PCR reaction mixture comprised 20 pM of each primer and in a total volume of 20 µL, including 5 µL of RNA template. The reverse transcription reaction was incubated at 45 °C for 30 min. The PCR amplification program consisted of an initial 5 min denaturation step at 94 °C, followed by 35 cycles of denaturation at 94 °C for 30 s, annealing at 55 °C for 30 s, and extension at 72 °C for 1 min and 30 s, with a 7-min final extension at 72 °C. Multiple negative controls were included to prevent cross-contamination during the nested PCR, along with a positive control provided by the Korea Disease Control and Prevention Agency. Amplified products were separated using electrophoresis on a 1.5% agarose gel and visualized under UV light. Positive samples were further analyzed using nucleic acid sequencing and identification.

### 2.4. Phylogenetic Analysis and NoV Genotyping

NoV identification was confirmed using nucleic acid sequence analysis. After electrophoresis, the nested RT-PCR products were excised from the gel using a razor blade and extracted using the QIAquick Gel Extraction Kit (Qiagen). Sequence analysis of the products was performed by Macrogen (Seoul, Republic of Korea). The obtained sequences were compared to those in the GenBank database using the NCBI BLAST search program. Phylogenetic analysis was conducted using the neighbor-joining method to determine the NoV genotype. Nucleotide sequences were analyzed against NoV reference sequences using MEGA-X software V.10.1.1 [18].

## 3. Results and Discussion

### 3.1. Groundwater Quality in South Korea

A total of 600 sampling sites were monitored from 2019 to 2023, with higher sampling frequencies in spring and winter than in summer and fall. NoV infection is often referred to as a “winter vomiting illness” and monitoring is increased during the winter months [15,19]. The distribution of sampling points in Figure 1 shows an even coverage across the country, indicating that groundwater is a widely used primary source of drinking water in most regions. The groundwater examined in this study primarily came from facilities over 40 years old, all of which were small-scale facilities with a daily capacity of 200 t. Table 1 summarizes the groundwater conditions and facilities at the sampling sites.

The average water temperature and turbidity levels decreased from 2019 to 2023. Residual chlorine levels remained relatively stable over the five years, with only slight fluctuations. A previous study reported an average water temperature of 16.4 °C, turbidity of 0.94 NTU, and residual chlorine levels of 0.58 ppm [9]. Additionally, a 2008 study of groundwater showed a water temperature of 19.46 °C and turbidity of 0.58 NTU [15]. Previous studies also reported an average groundwater temperature of 16.4 °C and 19.46 °C [9], respectively, turbidity of 0.94 NTU and 0.58 NTU, respectively, and residual chlorine levels of 0.58 ppm, similar to the findings of the current study.

Viral survival in the environment plays a crucial role in the infectivity of NoVs, with infectious doses as low as ten viral particles remaining viable for over two months [20]. Temperature is the most commonly reported environmental factor influencing NoV outbreaks. Studies using murine NoVs, a surrogate for human NoVs, have shown that these viruses can survive at 18 °C for up to 40 days [21]. Groundwater temperature is more stable than surface water [22], and the groundwater temperature ranged from 14.4 °C to 11.8 °C in this study, which may allow NoVs to persist in groundwater for extended periods. Additionally, the turbidity levels in groundwater are low owing to natural sand filtration, thereby enhancing water clarity [23].

### 3.2. Genotype and Characteristics of NoV-Positive Samples

Table 2 presents detailed results of NoV-positive samples collected from 2019 to 2023. Of the 600 samples, 11 (1.8%) tested positive for NoVs. The NoV GI primer sets were used to detect the GI.1 genotype in one case during spring and two cases during fall. The NoV GII primer sets were used to detect the GII.4 genotype in three cases during spring, two cases in summer, and three cases in winter. More GII strains were detected than GI strains during the five-year study period, and all GII samples were identified as GII.4 (spring: three; summer: two; winter: three). NoV GII.4 is the most prevalent and infectious form of the virus [24], and this genotype is the predominant cause of NoV infections in humans [25].

During NoV analysis, groundwater temperatures ranged from 5.5 °C to 21.3 °C, with the highest number (*n* = 7) of detections occurring between spring and winter (Table 2). The incidence of NoV increases seasonally from winter to spring and generally occurs between October and March [3].

Of the eleven NoV-positive samples, three had no detectable residual chlorine. In cases where residual chlorine was detected, the concentrations were generally low. Chlorine levels in drinking water should be maintained at 0.2–5.0 mg/L [26]. The USA EPA and South Korea’s Ministry of Environment have established guidelines for chlorine, a by-product of disinfection, at 4.0 mg/L in drinking water [27]. According to established standards, chlorine concentrations at the investigation sites were notably low [28]. Previous studies on the inactivation of murine NoV have indicated that chlorine is an effective chemical disinfectant. According to the U.S. EPA guidance manual for viral disinfection at 5 °C, a chlorine concentration of 8 mg/L·min with a Ct of 99.99 is recommended [29]. The results of this study suggest that groundwater should be managed to maintain residual chlorine concentrations at the recommended levels to ensure effective NoV removal.

Among the NoV-positive groundwater samples, total coliforms, a useful indicator of other pathogens in drinking water, were found in only three samples, while *Escherichia coli*, a fecal indicator bacterium, was not detected in any of the samples. In previous studies, only one variable, male-specific coliphages, showed a correlation with NoVs and microbial analysis, whereas no significant relationships were observed with other microorganisms [15]. However, the volumes of water analyzed for fecal indicators were much smaller than those analyzed for NoVs. Typically, smaller volumes of water are used for fecal indicator microorganism analyses because their concentrations are higher than those of pathogenic enteric viruses. These differences in the analyzed volumes may explain the absence of correlation coefficients [9]. Additionally, because fecal indicator microorganisms are generally larger than enteric viruses, soil filtration effectively filters these microorganisms, resulting in the reduced infiltration of fecal bacteria into groundwater, where they may exist at relatively low concentrations [30]. Therefore, in addition to other indicators, the analysis of fecal indicator viruses may be necessary for an accurate prediction of future NoV outbreaks.

### 3.3. Phylogenetic Analysis of NoV Genotypes

**Figure 2:**
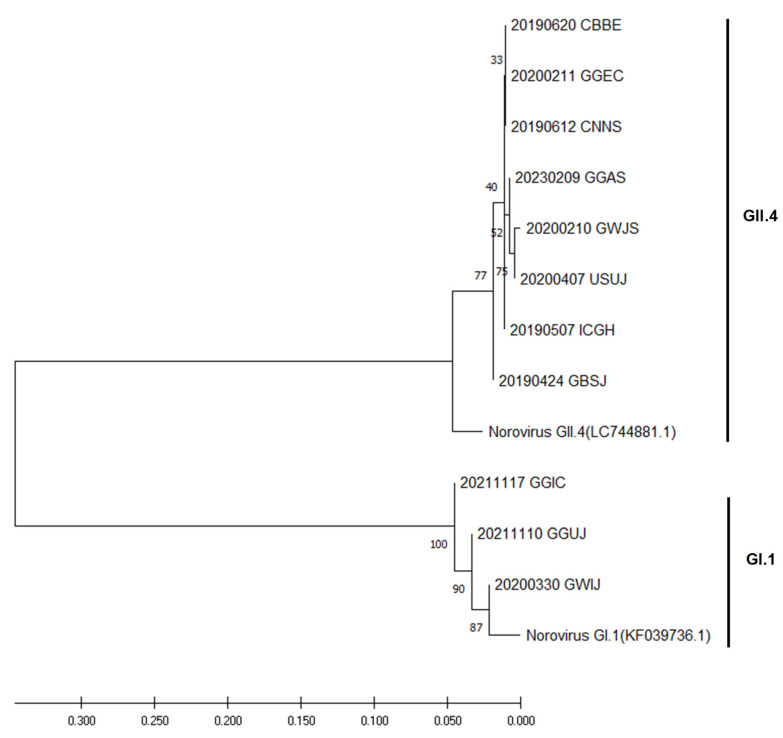
Phylogenetic analysis of norovirus genotypes in groundwater samples collected from 2019 to 2023. Bootstrap values from 1000 replicates are displayed to validate clustering.

The phylogenetic tree in Figure 2 illustrates the relationship between the NoV GII.4 and GI.1 genotypes identified from groundwater samples collected between 2019 and 2023. The analysis revealed two distinct clusters corresponding to the GII.4 and GI.1 genotypes, which were consistent with the reference sequences for these genotypes (LC744881.1 for GII.4 and KF039736.1 for GI.1). Most NoV-positive samples clustered under GII.4, which is the most prevalent genotype worldwide, particularly in outbreak settings. The bootstrap value for the branches of the samples collected between 2019 and 2023 was 77 within the GII.4 cluster, indicating high genetic similarity. Notably, the GBSJ sample collected on 24 April 2019 was identified as GII.4 and exhibited a close relationship with the reference strain NoV GII.4 (LC744881.1). These findings further indicate that GII.4 was the most frequently detected genotype across seasons nationwide and regionally. Similarly, other studies have shown that NoV GII.4 is the most widely detected genotype across various regions and periods [15].

The NoV GI.1 cluster demonstrated low genetic diversity, with bootstrap values ranging from 87 to 100, suggesting a stable viral population. This observation is consistent with those of previous studies, which have shown that the GI.1 genotype is less susceptible to genetic variation than GII.4 [31,32]. Furthermore, the close genetic relationship between the GI.1 sample and the reference strain NoV GI.1 (KF039736.1) reinforces the stability of this genotype over time and across regions. These results suggest that the NoV genotypes identified in groundwater may present a potential risk for future outbreaks, highlighting the importance of continued monitoring of GII.4 and GI.1 genotypes in environmental samples [32]. Ongoing phylogenetic surveillance is crucial for tracking the evolution of these genotypes and for providing valuable insights into the sources and transmission pathways of NoVs in the environment. However, due to the relatively small number of positive samples (11 out of 600), further analysis of a larger number of groundwater sites will be necessary to effectively track norovirus evolution. Increasing the number of sampling points in future studies will provide more comprehensive data and enhance our ability to monitor norovirus diversity and spread in groundwater sources.

## 4. Conclusions

This study investigated the prevalence and genotype of NoVs in groundwater from drinking water sources in South Korea. Eleven sampling sites were detected over five years and genotyped as GI.1 and GII.4. GII.4 was the most frequently detected. The detection of the highly infectious GII.4 variant in this study highlights the need for the continued monitoring of groundwater for public health. Given that groundwater management differs from the stricter national regulations applied to tap water, future efforts should include longer monitoring periods, widespread sampling, and detailed water quality assessments for effective management practices.

## Figures and Tables

**Table 1:** Summary of groundwater conditions and facilities (mean ± SD).

Year	No. of Sampling Sites (n)			Time of Facility Usage (years)	Daily Capacity (ton)	Water Temperature (°C)	Turbidity (NTU ^a^)	Residual Chlorine (ppm)
	Total	Winter/Spring	Summer/Fall					
2019	100	85	15	41 ± 10	61 ± 18	14.4 ± 4.3	1.09 ± 4.90	0.13 ± 0.26
2020	125	84	36	45 ± 4	64 ± 14	14.6 ± 5.2	0.6 ± 1.0	0.15 ± 0.40
2021	125	100	25	45 ± 60	116 ± 52	12.5 ± 4.5	0.71 ± 1.79	0.14 ± 0.27
2022	125	93	32	50 ± 6	73 ± 33	11.3 ± 4.4	0.58 ± 1.31	0.08 ± 0.14
2023	125	99	26	40 ± 13	75 ± 43	11.8 ± 4.3	0.44 ±0.54	0.09 ± 0.18

^a^ NTU: nephelometric turbidity unit.

**Table 2:** Water quality, genotype, and microorganism analysis of norovirus-positive samples.

Norovirus-Positive Samples						
Year	Genotype	Season	Water Temp (°C)	Residual Chlorine (ppm)	Total Coliform/100 mL	*Escherichia coli*/ 100 mL
2019	GII.4	Spring	14.8	0.75	P ^a^	ND ^b^
	GII.4	Spring	15.7	0.19	ND	ND
	GII.4	Summer	19.2	0.26	ND	ND
	GII.4	Summer	21.3	0.55	ND	ND
2020	GI.1	Spring	12.6	0.16	ND	ND
	GII.4	Spring	8.8	0.03	P	ND
	GII.4	Winter	5.5	0.02	ND	ND
	GII.4	Winter	9.1	0.34	ND	ND
2021	GI.1	Fall	15.5	0	ND	ND
	GI.1	Fall	14.3	0	ND	ND
2023	GII.4	Winter	11.3	0	P	ND

^a^ P: Present. ^b^ N: Not Detected.

## Data Availability

The data presented in this study are available from the corresponding author on reasonable request.
